# Niche Partitioning of Feather Mites within a Seabird Host, *Calonectris borealis*

**DOI:** 10.1371/journal.pone.0144728

**Published:** 2015-12-09

**Authors:** Laura M. Stefan, Elena Gómez-Díaz, Eric Elguero, Heather C. Proctor, Karen D. McCoy, Jacob González-Solís

**Affiliations:** 1 Institut de Recerca de la Biodiversitat (IRBio) and Departament de Biologia Animal, Universitat de Barcelona, Barcelona, Spain; 2 Estación Biológica de Doñana, Consejo Superior de Investigaciones Cientificas (CSIC), Isla de La Cartuja, Sevilla, Spain; 3 MIVEGEC Research Unit, UMR 5290, CNRS-IRD-UM, Centre IRD, Montpellier, France; 4 Department of Biological Sciences, University of Alberta, Edmonton, Alberta, Canada; Universidad de Granada, SPAIN

## Abstract

According to classic niche theory, species can coexist in heterogeneous environments by reducing interspecific competition via niche partitioning, e.g. trophic or spatial partitioning. However, support for the role of competition on niche partitioning remains controversial. Here, we tested for spatial and trophic partitioning in feather mites, a diverse and abundant group of arthropods. We focused on the two dominant mite species, *Microspalax brevipes* and *Zachvatkinia ovata*, inhabiting flight feathers of the Cory’s shearwater, *Calonectris borealis*. We performed mite counts across and within primary and tail feathers on free-living shearwaters breeding on an oceanic island (Gran Canaria, Canary Islands). We then investigated trophic relationships between the two mite species and the host using stable isotope analyses of carbon and nitrogen on mite tissues and potential host food sources. The distribution of the two mite species showed clear spatial segregation among feathers; *M*. *brevipes* showed high preference for the central wing primary feathers, whereas *Z*. *ovata* was restricted to the two outermost primaries. Morphological differences between *M*. *brevipes* and *Z*. *ovata* support an adaptive basis for the spatial segregation of the two mite species. However, the two mites overlap in some central primaries and statistical modeling showed that *Z*. *ovata* tends to outcompete *M*. *brevipes*. Isotopic analyses indicated similar isotopic values for the two mite species and a strong correlation in carbon signatures between mites inhabiting the same individual host suggesting that diet is mainly based on shared host-associated resources. Among the four candidate tissues examined (blood, feather remains, skin remains and preen gland oil), we conclude that the diet is most likely dominated by preen gland oil, while the contribution of exogenous material to mite diets is less marked. Our results indicate that ongoing competition for space and resources plays a central role in structuring feather mite communities. They also illustrate that symbiotic infracommunities are excellent model systems to study trophic ecology, and can improve our understanding of mechanisms of niche differentiation and species coexistence.

## Introduction

A niche can be defined as the global environmental requirements of a species to complete its life cycle, and includes its impact on resource availability and on other organisms in the community [1]. According to classic niche theory, species can coexist in heterogeneous environments by reducing interspecific competition via niche partitioning [2,3]. Different types of niche partitioning can occur, such as spatial niche partitioning, when species share a food resource but use distinct subsets of the habitat, and trophic partitioning, when different species specialize on distinct food resources in sympatric habitats [4].

However, support for the role of competition on niche partitioning remains controversial. Observational studies quantifying static patterns among co-occurring species are difficult to interpret unequivocally [5,6]. A common problem is the lack of sufficient replicates that limits the detection and analysis of general patterns of community structure. In this regard, permanent obligate symbionts (commensals, mutualists or parasites) have been proposed as good models for understanding community structure and the dynamics of niche partitioning over small spatial scales. In these systems spatial and trophic resources are limited to the body of the host and each host represents a replica of a discrete habitat patch [7].

Intrinsic host factors along with extrinsic environmental factors can influence the distribution of obligate symbionts on or within a host, but structuring can also arise due to direct interaction among species [8]. Indeed, interspecific competition is considered to be a major process shaping symbiont infracommunities [7]. Spatial segregation has been examined for both endo- and ectosymbionts of fish, birds and mammals [9–12], but evidence from different studies is inconsistent, with some studies supporting a role for competition and others suggesting a tendency for co-occurring symbionts to aggregate in preferred areas of the host body [13].

Feather mites (Astigmata: Pterolichoidea, Analgoidea) are the most diverse obligate ectosymbionts living on birds [14] and have been reported from all avian orders with the exception of Rheiformes [15]. They do not have an off-host stage and are transferred by direct contact between mates, parents and offspring, and potentially other flock members if there is close contact (e.g., fighting or flock feeding). In contrast to skin-dwelling mites and feather lice, which are often transmitted horizontally among hosts by hippoboscid flies [16,17], there have been very few observations of such indirect transmission of feather-dwelling mites [18]. Previous studies have shown that these species often show marked differences in distribution among feathers, with some being restricted to certain feather types or regions within a feather [12,19–21]. Although the distribution of feather mites are at least partially related to specific habitat and trophic morphological specializations (e.g. body shape, setae, structure of the mouthpats) [14,22,23], the role of resource competition as a mechanism generating this diversity is largely unknown.

To date, a number of studies have investigated the spatial distribution of feather mites on individual hosts [12,19,21,24,25], but few have evaluated their trophic relationships and the nature of their ecological interactions with the host. Part of the difficulty in studying mite ecological relationships is due to their small size and the inability to maintain them off of the bodies of their normal hosts. Although some authors provide evidence that feather mites are parasites, causing damage to their hosts [26,27], most studies suggest that they are commensals living on the surface of host feathers [28,29]. Based on the morphological structure of the mouthparts and observations of the guts of slide-mounted mites, it has been suggested that feather mites feed principally on oil produced by the uropygial gland, and on debris trapped between the feather barbs such as fungal spores and pollen grains [30–32]. Skin remains and feather fragments have also been occasionally observed in mite guts but are common only in some species [14]. Indirect methods, such as stable isotope analyses (SIA), can be powerful tools for studying trophic relationships of otherwise difficult to observe organisms [33]. This approach is based on the fact that isotopic signatures of different dietary sources are reflected in the tissues of consumers in a predictable manner [34]. Nitrogen (^15^N/^14^N) is typically used to infer the trophic position of consumers, and increases by approximately 2.5‰-5‰ with each trophic level [35], whereas carbon (^13^C/^12^C) is typically used to describe the diet sources, and shows only a limited enrichment between trophic levels (0–1‰) [36]. SIA has been successfully applied to study trophic interactions in different host-parasite systems including both endoparasites, such as intestinal nematodes and cestodes [37,38] and ectoparasites, such as lice, fleas and bat flies [39,40], but to the best of our knowledge has not been used to study mites.

Here, we examine the spatial organization and trophic structure of the two principal feather mite species inhabiting flight feathers of the Cory´s shearwater, *Calonectris borealis* (Cory) (Procellariiformes: Procellariidae). Our specific objectives were (1) to assess the occurrence of niche partitioning by examining relative within-host distribution and resource use of the two mite species, and (2) to test the extent of the spatial competition, that is, whether the distribution and abundance of one mite species limits the distribution and abundance of the other. If niche partitioning occurs, we expected that the two species would either (a) share the same food resource (i.e., share a common isotopic signature), but occupy distinct and non-overlapping regions of the host’s body, (b) consume different foods (i.e. have different isotopic signatures) and occupy the same parts of the host or (c) segregate in both trophic resources and space use. These hypotheses are consistent with competition playing an important role in determining niche partitioning of these mites, but could also result from independent microhabitat adaptation. To explore the role of ongoing competition in determining mite distributions, we investigated changes in occupancy patterns among individual hosts. If one mite species actively excludes the other, we expected a shift in distribution and abundance when the competing species is present. If the distribution and abundance of one species does not affect that of the other but the two remain spatially segregated even in the absence of potential competitors, we considered spatial segregation to result from an independent adaptive process or from selective pressure from past competition (i.e. the ghost of competition past).

## Materials and Methods

### Study area and species

Our study focused on a population of Cory´s shearwater breeding in the location of Veneguera, Gran Canaria, Canary Islands (27°50^’^N, 15°47^’^W). The Cory´s shearwater breeds mainly in the northeast Atlantic Ocean, from the Canary to the Azores Archipelagos and hosts a wide array of ectosymbionts, such as lice, fleas, and ticks, along with at least six described species of feather mites: *Microspalax brevipes*, *Microspalax ardennae*, *Zachvatkinia ovata*, *Rhinozachvatkinia calonectris*, *Promegninia calonectris* and *Ingrassia calonectris* [41–46]. Fieldwork was carried out during the Cory’s shearwater breeding season, from mid June to mid July 2011 and a total of 60 birds were captured and examined at night.

### Feather mite counts

**Fig 1:**
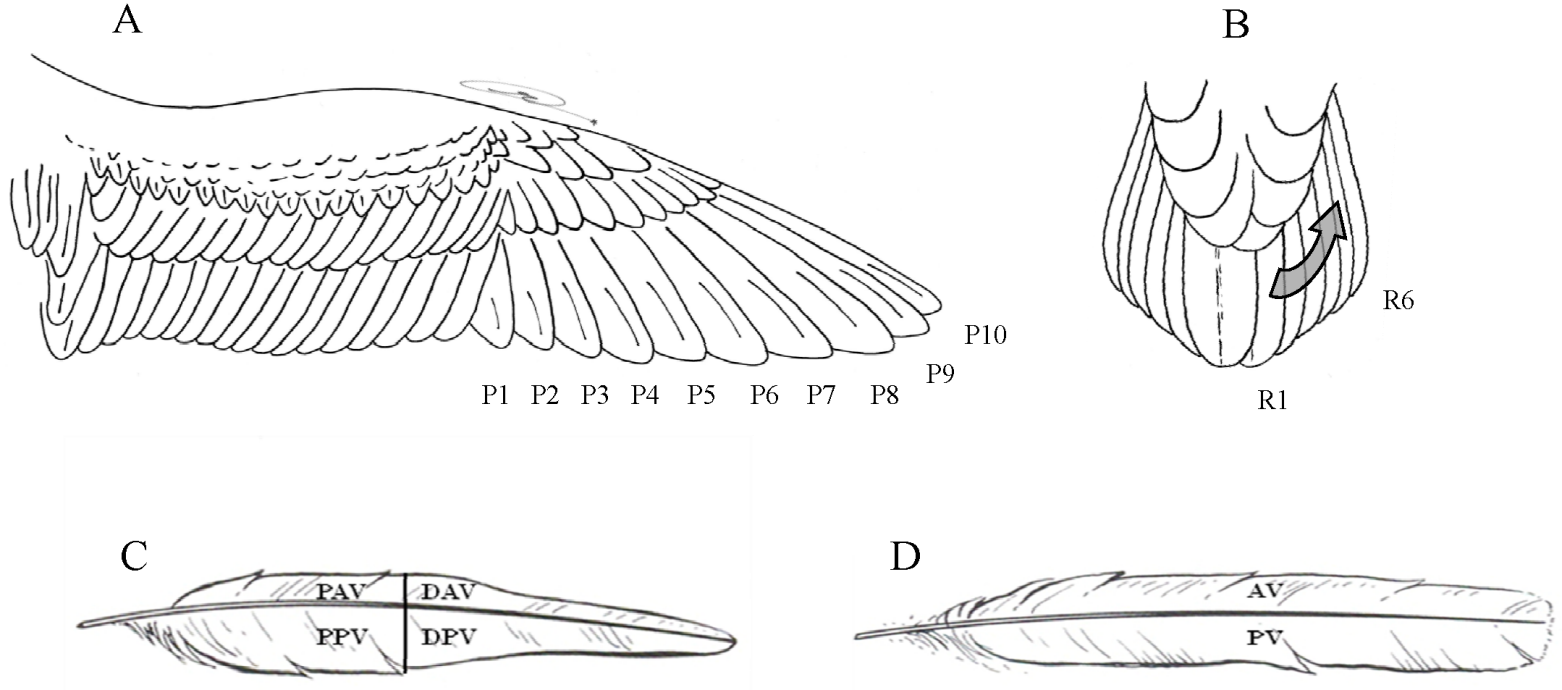
**Primaries (A) and rectrices (B) of Cory´s shearwater** (see [58]). Primaries were divided into four equal regions: PPV—proximal posterior vane, DPV—distal posterior vane, PAV—proximal anterior vane, DAV—distal anterior vane (C), while rectrices were divided into two regions: AV—anterior vane and PV—posterior vane (D).

We performed mite counts directly on the birds in the field. In order to have sufficient and reliable data for each individual, we focused counts on the two most abundant feather mite species, *M*. *brevipes* (*Mb*) and *Z*. *ovata* (*Zo*), inhabiting flight feathers (primaries and rectrices) (Fig 1A and 1B). Other mite species were also observed on flight feathers, but they were occasionally found and were relatively difficult to distinguish at low magnification in the field, due to their poor pigmentation and their smaller body sizes compared to *Mb* and *Zo*. The two species are easily distinguishable from each other at low magnification. *Mb* male and female have heavily sclerotized ovoid bodies, short, broad forelegs and ventrally inserted hind legs and both sexes look similar to the unaided eye. *Zo* is 1.5 to 2 times longer than *Mb* and has long forelegs and laterally inserted hind legs. The posterior margin of *Zo* female body is rounded, whereas that of the male bears two terminal lobes. Adults of both species are darkly pigmented and so they stand out clearly against the light-coloured feathers, while juvenile mites are often too poorly pigmented to be reliably counted; we thus restricted the analysis to adults. For each bird, we counted the number of adult *Mb* and *Zo* present on the ten primary feathers (P1-P10) and on the six rectrices (tail flight feathers) (R1-R6) of the left side, using a 10X hand magnifier (S1 Table). Each primary feather was divided into four approximately equal regions (Fig 1C): proximal anterior vane (PAV), distal anterior vane (DAV), proximal posterior vane (PPV) and distal posterior vane (DPV), while rectrices were only divided into two regions, anterior (AV) and posterior (PV) vane, because of the relatively small number of feather mites found on these feathers (Fig 1D). In the case of P10, we observed in the field that the posterior region of the feather is unsuitable for mites due to structural features, and mites were therefore not counted in the DAV and PAV regions of this feather. To reduce handling time and associated stress on the bird, when more than 100 adult mites for one species were observed in a region, the count for this region was assigned to the category “>100”.

To confirm the accuracy of the counting method used, we repeated counts on the same individual bird after one or two days, for a subset of nine birds. We then calculated the Intraclass Correlation Coefficient (ICC) [47] using a one-way random model for the total number of mites for each species and for the number of mites per feather. All counts of “>100 mites” were assigned an arbitrary value of 150.

### Distribution of mites among and within feathers

Among flight feathers: To assess potential interactions between the two mite species on flight feathers, we first computed the total mite count per feather. For this analysis, the truncated counts (those assigned to the “>100” category) were replaced by randomly selected values between 100 and 200, as counts only very rarely exceeded 200 mites per region. We then used generalized linear mixed models (GLMM) [48] to explain the count of a given feather mite species by three fixed-effect covariates: i) the log-transformed count of the other mite species (log2(1+count)); ii) host sex and iii) the relative position of the feather in the wing, here defined as proximal: P1-P3, intermediate: P4-P7 and distal: P8-P10. The host individual was introduced as a random factor. The distribution of counts was assumed to be a zero-inflated negative binomial. Since this analysis depended on random values for the truncated counts, it was repeated 500 times, thus yielding 500 coefficient values and 500 P-values. In the results, we reported the average model values obtained for each mite species. The model estimations were performed using the R package glmmADMB [49]. The same analysis was applied to data from the rectrices, which were classified into only two relative positions, proximal (R1-R3) and distal feathers (R4-R6).

Among feather regions, within a primary feather: To analyze the interaction between the two mite species at the within-feather scale, we restricted our analysis to the presence/absence of each mite species within a feather region. For this purpose, we used a mixed binomial model, where the probability of presence of *Mb* in a given feather region was explained by the presence of *Zo* mites in that same region, the position of the region on the feather (PAV, DAV, PPV or DPV), plus a host random effect. In this analysis, we considered only primaries P6 to P9, where both species were potentially present. The result was reported as an odds-ratio, where a ratio smaller than 1 indicates a negative interaction between the two species.

### Niche breadth and niche overlap

Niche breadth and overlap of the two mite species were measured at two different spatial scales using Levins’ equations as described by Choe and Kim [19]. First, in order to examine interactions between co-occurring mite species along the entire wing, all primaries were treated as a data set and individual primaries were considered as states. Second, to describe the niche relationships between co-occurring mite species on different parts of the primary feather, the four feather regions were treated as a dataset and individual regions as states. An arbitrary value (150) was assigned for all “>100” counts. The values of niche breadth (B) and niche overlap (O) theoretically range from 0 to 1. However, the calculated value may exceed 1 when the broader-niched species has a larger carrying capacity [50]. Although there are no critical levels with which overlap values can be compared, it has been suggested that values higher than 0.6 should be considered as biologically significant [51].

### Stable isotope analyses (SIA)

For SIA analyses, we sampled small fragments of feather barbs containing mites from 20 birds. The two mite species were sampled from the primaries where they were most abundant: P4, P5 or P6 for *Mb* and P9 or P10 for *Zo*. Mites from each individual feather were removed from the barbs, identified and separated by species into pools ranging from 30 to 226 individuals for *Mb* and from 18 to 164 individuals for *Zo*. The pools were then dried in an oven at 45°C for 6 hours, weighed and placed into ultra-clean tin capsules. Sample mass of each mite pool ranged from60 to 200 μg for *Mb* and from 60 to 230 μg for *Zo*, except for three samples for *Mb* and one sample for *Zo* with a low number of mites, resulting in a sample mass ranging from 32 to 58 μg. Barbs were washed in a 0.25 M sodium hydroxide solution, rinsed thoroughly in distilled water to remove any surface contamination, dried in an oven at 45°C to a constant mass and cut into small pieces manually. From the sampled birds, 0.5 ml of blood was also collected and preserved at -20°C. Host blood was lyophilized for 24 hours using a Telstar Cryodos-50 freeze-dryer and then ground into powder manually. Samples ranging from 300 to 320 μg of blood powder or of feathers were weighed and placed into ultra-clean tin capsules. Fourteen samples of preen gland oil and 13 of wing skin were also analyzed. These samples were taken from dead frozen Cory´s shearwaters from the same island location that had been euthanized at the Recovery Wildlife Center Tafira (Gran Canaria) due to bone fractures. An incision was made in the uropygial gland and the contents were preserved in vials at -20°C. For skin samples, feathers were removed from a small area of the wing at the junction between the humerus and ulna and a sample of epidermis was removed with a scalpel and forceps. Subsequently, all uropygial and skin samples were treated as host blood. Lipids are usually extracted from lipid rich tissues before SIA since it has been shown that lipids are depleted in δ13C values [52]. However, we did not extract lipids from the preen gland oil because this tissue is basically only composed of lipids and it is thought that mites can feed on these secretions. Sample mass ranged from 285 to 335 μg for uropygial gland secretions and from 250 to 300 μg for wing skin. All samples were oxidized in a Flash EA1112 Elemental Analyzer and a pirolizator TC-EA coupled to a Delta C Finnigan MAT mass spectrometer through a Conflo III interface (ThermoFinnigan), where δ^13^C and δ^15^N signatures were determined (Isotopic ratio mass spectrometry, Serveis Científico-Tècnics of University of Barcelona, Spain). Isotope ratios were expressed conventionally as δ values in ppt (‰) according to the following equation:

$$\delta X=\;\left[(R_{\text{sample}}/R_{\text{standard}}\right)\;–\;1]\;\times \;1000$$

where *X* (‰) is ^13^C and ^15^N, and *R* are the corresponding ratios ^13^C/^12^C and ^15^N/^14^N, related to the standard values: *R*_standard_ for ^13^C is Vienna Pee Dee Belemnite (VPDB), for ^15^N is atmospheric nitrogen (AIR) (S2 Table). International standards (IAEA CH_7_ and IAEA CH_6_ for C, IAEA N_1_ and IAEA N_2_ for N, USGS 34, USGS 40 and acetanilide for both C and N) were run every 12 samples to calibrate the system and compensate for any drift over time. Replicate assays of standard materials indicated measurement errors of ±0.1 and ±0.2‰ for carbon and nitrogen respectively, but these are likely underestimates of true measurement error for complex organics like feathers and mite tissues.

The statistical analyses for mite stable isotopes were performed using SPSS 15.0 for Windows (IBM SPSS Statistics). To test for differences in stable isotopic values among mite species and host tissues (blood and feathers), we applied a linear mixed model (LMM) using the restricted maximum likelihood (REML) estimation method. The type of tissue (*Mb*, *Zo*, host blood and feathers) was treated as a fixed factor and host identity as a random term. Bonferroni corrections on post-hoc comparisons were performed. Preen gland oil and wing skin were not included in the LMM analysis because these tissues were not isolated from the same living birds; however, the mean values for all host tissues and mites and their 95% confidence intervals were visually compared.

### Ethics statements

This present work was carried out in a single location, Veneguera, Gran Canaria, Canary Islands and the permits to capture and examine live procellariiform birds were issued by Cabildo Insular de Gran Canaria (authorization n°1169/2011) and Gobierno de Canarias (authorization n° 0795/2011). No other locations were sampled for which specific permission was not required. Fieldwork involved handling a protected seabird species, the Cory’s shearwater (*Calonectris borealis*), for which we obtained the corresponding permission. Birds were captured by night in their nests. We sampled small fragments of feather barbs containing mites from primaries P4, P5 or P6 and P9 or P10 and 0.5 ml of blood from the tarsal vein, using a 1 ml syringe, from 20 birds. All procedures were approved by local (Cabildo Insular de Gran Canaria) and regional (Gobierno de Canarias) authorities and no approval was obtained from any animal ethics committee because authorities did not consider it necessary. All sampling procedures were specifically approved as part of obtaining the field permits. Samples of preen gland oil were taken from dead frozen Cory´s shearwaters obtained from the Recovery Wildlife Center Tafira (Gran Canaria). These birds had been euthanized due to bone fractures.

## Results

### Infestation and repeatability

All but one of the 60 captured birds harboured feather mites on the flight feathers from the left side of the body. From the 59 birds with mites, four were infested only with *Microspalax brevipes* (*Mb*), while the remaining 55 birds had both *Mb* and *Zachvatkinia ovata* (*Zo*). Usually, mites were located along the length of the rachis, at the base of the barbs, but in heavily laden host individuals, mites also occupied the ventral surfaces of the barbs distal to the rachis.

The repeatability analysis, based on the total number of mites, indicated that the two mite species counts were highly correlated (ICC = 0.983; F_[142,143]_ = 113.32; P < 0.001 for *Mb* and ICC = 0.956; F_[142,143]_ = 44.6; P < 0.001 for *Zo*). When considering counts per feather, there was still a significant correlation, but the relationship was weaker (ICC ranging from 0.877 to 1 out of 32 tests corresponding to each primary and tail feather for the two mite species, all P < 0.001 for *Mb* and from 0.683 to 1, P < 0.05 for *Zo*). Thus, this analysis confirms the validity of our approach to assess spatial distribution of feather mites.

### Spatial distribution of feather mites

**Fig 2:**
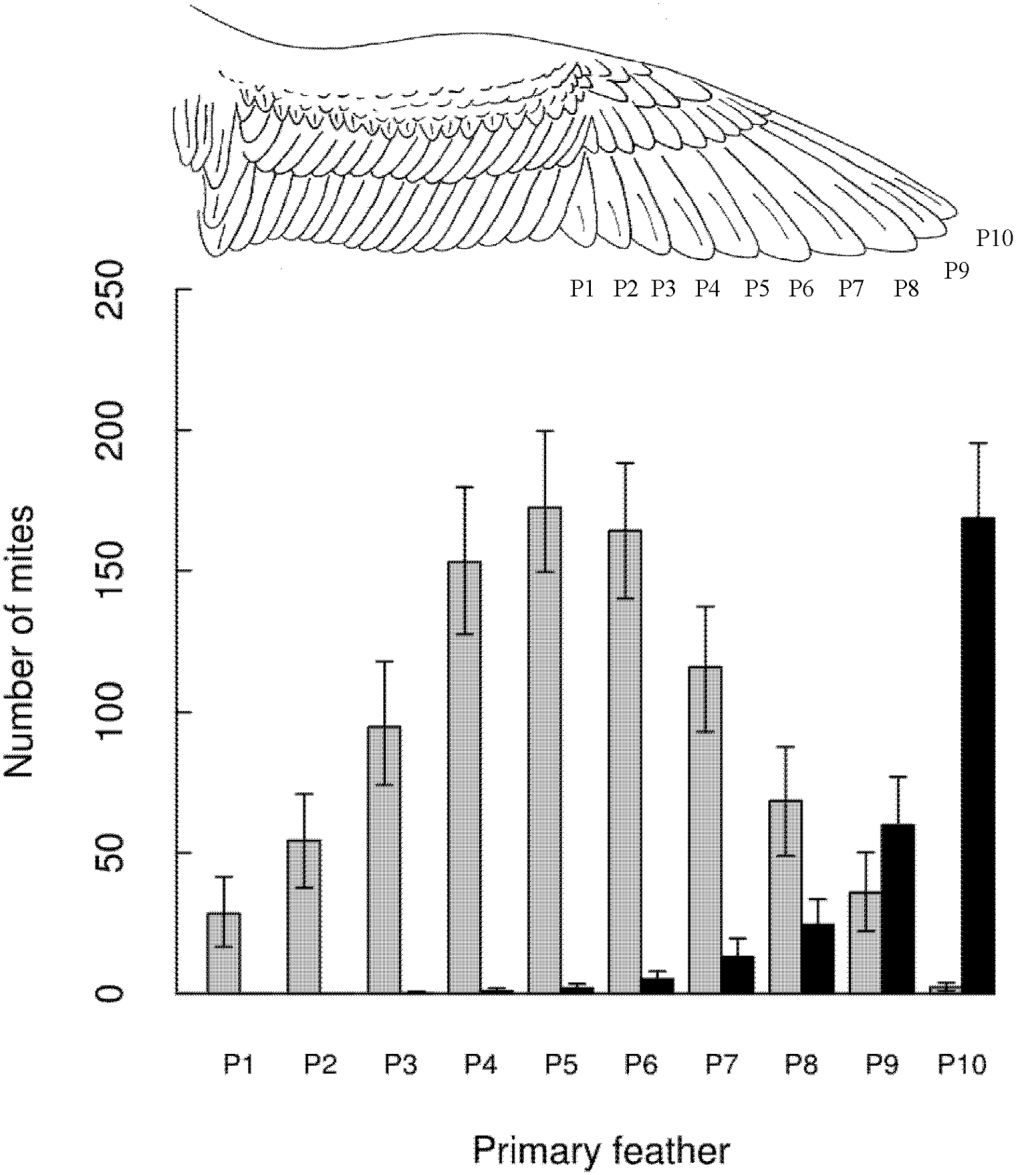
Distribution of *Mb* (gray) and *Zo* (black) in the ten primaries of Cory´s shearwater left wing. Feathers are ordered following their position in the wing from internal (P1) to external (P10) primary feathers. “Number of mites” represents the mean number of mites of each species per feather. The 95% confidence limits were computed by resampling using 500 bootstrapped values.

Among feathers: The two mite species showed differences in their distribution patterns among primary (P) feathers (Fig 2). *Mb* was mainly concentrated on the central primaries (P3-P7), whereas *Zo* showed its highest abundance on the outermost two primaries (P9-P10). However, the two species overlap on a number of distal primaries (from P6 to P9, Fig 2). If we consider the four birds that harboured only *Mb*, the distribution of this mite species was slightly displaced towards the tip of the wing, with the highest peaks reached on the P5-P7 feathers, but with almost no mites occupying the outermost primary feather (P10) (S1 Fig). Rectrices were occupied mainly by *Mb*, but in low numbers compared with primary feathers (S2 Fig). Only two specimens of *Zo* were found on the rectrices of two birds whose presence can be considered accidental.

The GLMM analysis indicated significant differences in the average proportion of mites among feathers. The model used to determine the average coefficient is a multiplicative one. Thus, there were 2.98 times more *Mb* on intermediate than on proximal primaries (average P-value < 0.001) and 1.61 times more *Mb* on distal than on proximal primaries (average P-value = 0.003). Counts of *Mb* were negatively associated with counts of *Zo* (average coef = 0.495; average P < 0.001) meaning an average drop of 50.5% in the counts of *Mb* with each doubling of *Zo* abundance. For *Zo*, only intermediate and distal primaries were considered in the analysis, as *Zo* was exceedingly rare on proximal primaries. In this case, differences were also significant *Zo* being 9.5 times more abundant on distal compared to intermediate primary feathers (average P-value < 0.001). As above, the relationship between the counts of *Zo* and *Mb* was negative (average coef = 0.555; average P < 0.001), indicating an average drop of 49.5% in the counts of *Zo* for each doubling of *Mb* abundance. On rectrices, the GLMM analysis showed no significant differences in mite numbers between the proximal and distal groups (coef = 0.947; P = 0.53). The effect of host sex was not significant for either primaries (average coef = 1.86; average P = 0.18 for *Mb* counts; average coef = 1.30; average P = 0.66 for *Zo* counts) or rectrices (*Mb* alone, coef = 1.16; P = 0.72).

**Fig 3:**
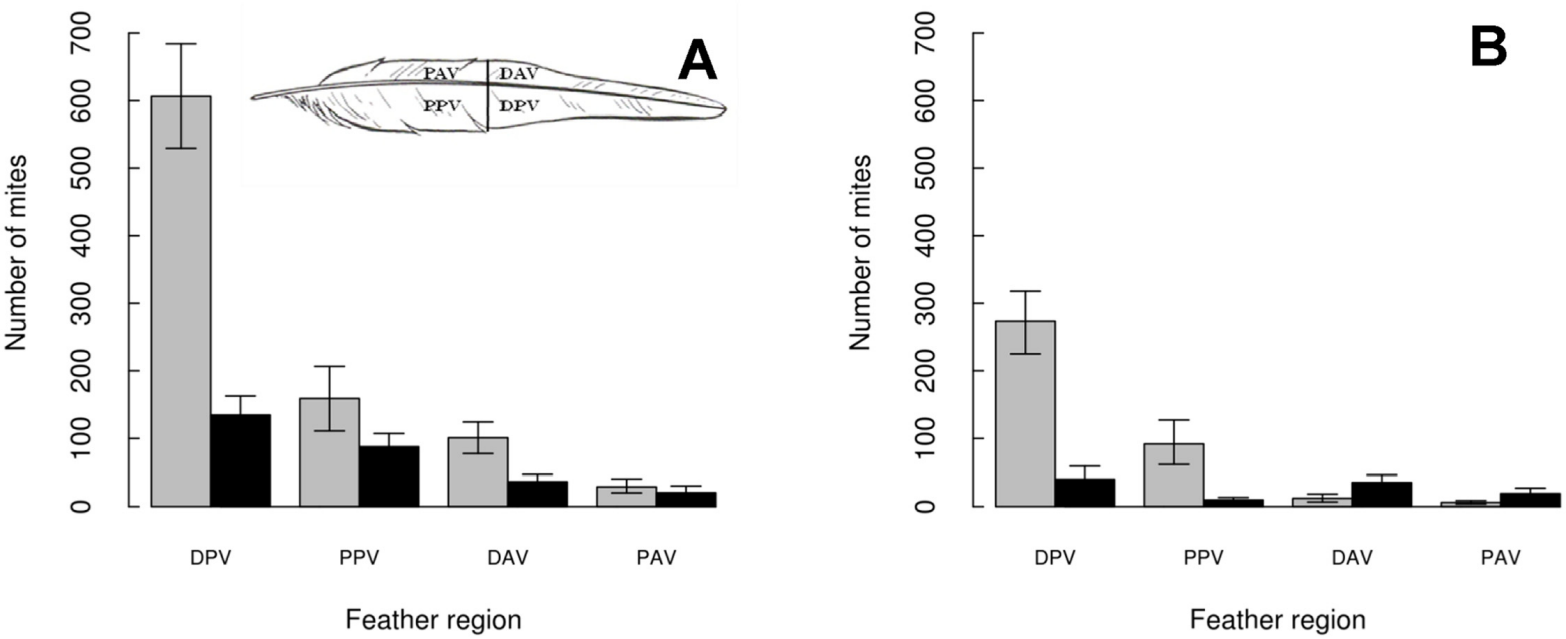
Mean number of mites and 95% confidence interval of *Mb* (grey) and *Zo* (black) across the four regions of all Cory´s shearwater primary feathers (A) and on P6-P9 feathers, where the two mite species co-occur (B). The “number of mites” represents the mean number of mites of each species per feather region. DPV = distal posterior vane; PPV = proximal posterior vane; DAV = distal anterior vane; PAV = proximal anterior vane.

Within feathers: Both mite species presented similar distributions among the four regions of the ten primaries, showing a clear preference for the posterior vane, in particular for the distal portion of the posterior vane (DPV), and avoiding the proximal anterior vane (PAV) region (Fig 3A). However, some spatial segregation arose where the two mite species co-occurred (P6-P9) (Fig 3B). In general, there was a tendency for a decreased abundance of the two species when both were present in the same region. By region, the decrease was more marked for *Zo* with respect to *Mb* in the proximal posterior vane region (PPV) and more marked for *Mb* with respect to *Zo* in both the distal (DAV) and the proximal anterior vane (PAV) regions. Overall, the odds-ratio for the presence of *Mb* according to the presence or absence of *Zo* was 0.41 (P < 0.001), indicating a reduction of the probability of *Mb* being present on a given feather region if *Zo* was also present on the same feather region.

### Niche breath and overlap

To test spatial niche differentiation between the two mite species, we first treated primary feathers as binary states. The niche breadth of *Mb* (B_Mb_ = 0.706) was found to be more than three times larger than that of *Zo* (B_Zo_ = 0.228). Overall, the niche spatial overlap was low between the two feather mite species, but with some asymmetry, so, the niches of *Mb* overlapped with *Zo* (O_MbZo_ = 0.208) to a greater extent than the reverse (O_ZoMb_ = 0.067). In contrast, when we reduced the spatial scale and considered the four feather regions within primaries as states, the niche breadth of *Zo* (B_Zo_ = 0.699) was found to be greater than that of *Mb* (B_Mb_ = 0.497). Within a feather, the niches of the two species overlapped significantly (O_MbZo_ = 0.804 and O_ZoMb_ = 1.131). When the same analyses were restricted to the range of primary feathers where the two species usually overlap, that is from P6 to P9, the within-feather niche breadth of *Zo* (B_Zo_ = 0.827) was also found to be greater than that of *Mb* (B_Mb_ = 0.436), and the two species also overlapped significantly (O_MbZo_ = 0.538 and O_ZoMb_ = 1.020), but there was a decrease in the overlap of *Mb* with *Zo* compared to the overlap when all primaries were considered (Fig 3A and 3B). These results suggest *Mb* specializes more on feather regions than on particular feathers, whereas *Zo* shows stronger affinities for particular feathers with a large use of feather regions.

### Trophic relationships

Isotopic values for all tissues (mites, host blood, feathers, preen gland oil and wing skin) did not depart from normality for both ^15^N and ^13^C (Kolmogorov-Smirnov test, all P > 0.05), except host blood and feathers for ^13^C (P < 0.010).

**Fig 4:**
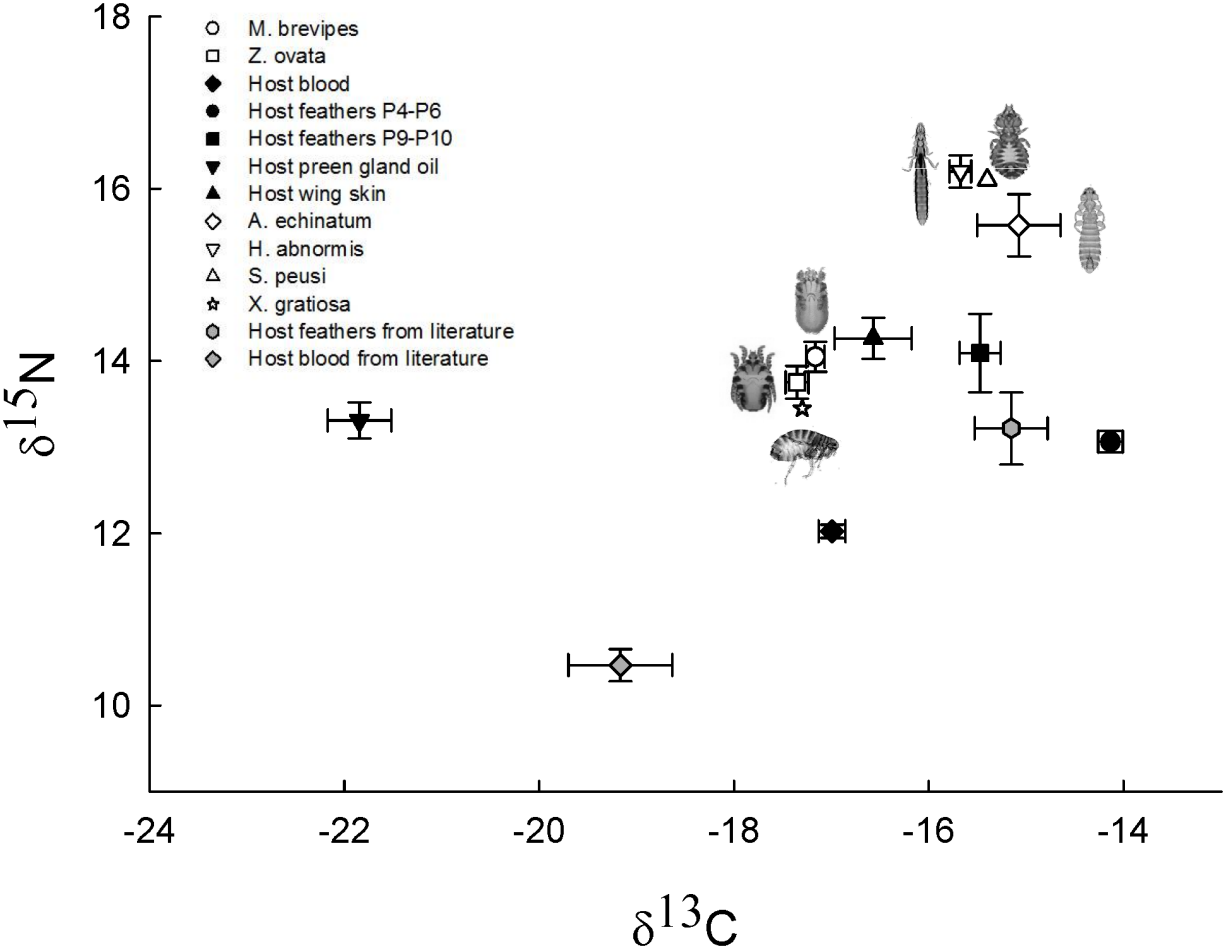
Mean δ^13^C and δ^15^N isotopic values of feather mites (*Mb* and *Zo*) and host tissues (blood, feathers, preen gland oil and wing skin) from Cory´s shearwater breeding in Veneguera. Preen gland oil and wing skin were isolated from dead birds belonging to the same species and same island location. *Mb* was sampled from P4-P6 feathers and *Zo* from P9-P10. Mean δ^13^C and δ^15^N isotopic values of other ectoparasite species (three louse species: Austromenopon echinatum, Halipeurus abnormis, Saemundssonia peusi and one species of flea: Xenopsylla gratiosa) and host tissues (blood and feathers) from Cory´s shearwater taken from Gómez-Díaz and González-Solís 2010 were also included. Error bars represent standard error. For X. gratiosa and S. peusi the error bars are not shown because of the small number of samples (n = 2 and n = 1, respectively). Isotopic values were not corrected for fractionation.

To investigate feeding preferences of mite ectosymbionts in relation to their hosts, we applied linear mixed model analyses (LMM) to compare the carbon and nitrogen stable isotope values of feather mites and host tissues. LMM showed that stable isotope values differed significantly among tissues (blood, feather and mite species) in both nitrogen (F_4, 75.37_ = 17, P < 0.001) and carbon (F_4, 75.35_ = 119.674, P < 0.001) values. Both mite species showed similar mean δ^13^C and δ^15^N values (Table 1, Fig 4) but no significant differences in nitrogen and carbon were found (D = 0.252, df = 75.195, P = 1.00 for nitrogen; D = 0.189, df = 75.177, P = 1.00 for carbon). Host blood showed the lowest mean value in nitrogen (12.022 ± 0.078), while *Mb* and feathers P9-P10 showed the highest (14.044 ± 0.176 and 14.039 ± 0.444, respectively) (Table 1, Fig 4). Furthermore, nitrogen comparisons among host blood and all other type tissues, including the two feather mite species and the host feathers, were all significant (*Mb*: D = −2.022, P < 0.001; *Zo*: D = −1.771, P < 0.001; feathers P4-P6: D = −1.040, P = 0.007; feathers P9-P10: D = −2.019, P < 0.001). Regarding carbon, the two feather mite species presented the lowest mean values (*Zo*: −17.354 ± 0.118; *Mb*: −17.165 ± 0.094), while feathers P4-P6 exhibited the highest mean value (−14.136 ± 0.126) (Table 1). We found significant differences between values of the two species of feather mites and their corresponding host feathers (*Mb*: D = −3.029, P < 0.001; *Zo*: D = −1.884, P < 0.001), but not between mites and the host blood (*Mb*: D = −0.171, P = 1.00; and *Zo*: D = −0.360, P = 0.477). Given that the preen gland oil and wing skin were isolated from dead birds, these two host tissues were not included in the linear mixed model analyses. However, preen gland oil presented the lowest mean δ^13^C value of all tissue types (−21.845 ± 0.325), including the feather mites, whereas wing skin showed the highest δ^15^N value (14.262 ± 0.239) (Table 1, Fig 4).

**Table 1:** Mean and percentage of carbon and nitrogen stable isotope values for the two feather mite species and host tissues (feathers, blood, preen gland oil and wing skin), from Cory´s shearwaters breeding in Veneguera. Values report mean and standard error (n = number of analyzed samples).

Mean C and N				Percentage of C and N	
	N	δ^13^C(‰)	δ^15^N(‰)	%C	%N
M. brevipes(Mb)	20	-17.165±0.094	14.044±0.176	49.160±1.889	10.399±0.440
Z. ovata (Zo)	20	-17.354±0.118	13.792±0.188	48.704±0.384	10.448±0.129
P4-P6	20	-14.136±0.126	13.062±0.125	47.475±0.101	14.878±0.052
P9-P10	19	-15.473±0.210	14.039±0.444	47.702±0.126	15.053±0.056
Blood	20	-16.994±0.137	12.022±0.078	47.203±0.437	13.997±0.133
Preen gland oil	14	-21.845±0.325	13.309±0.208	70.102±0.970	3.745±0.326
Wing skin	13	-16.571±0.395	14.262±0.239	50.455±0.802	13.995±0.421

**Fig 5:**
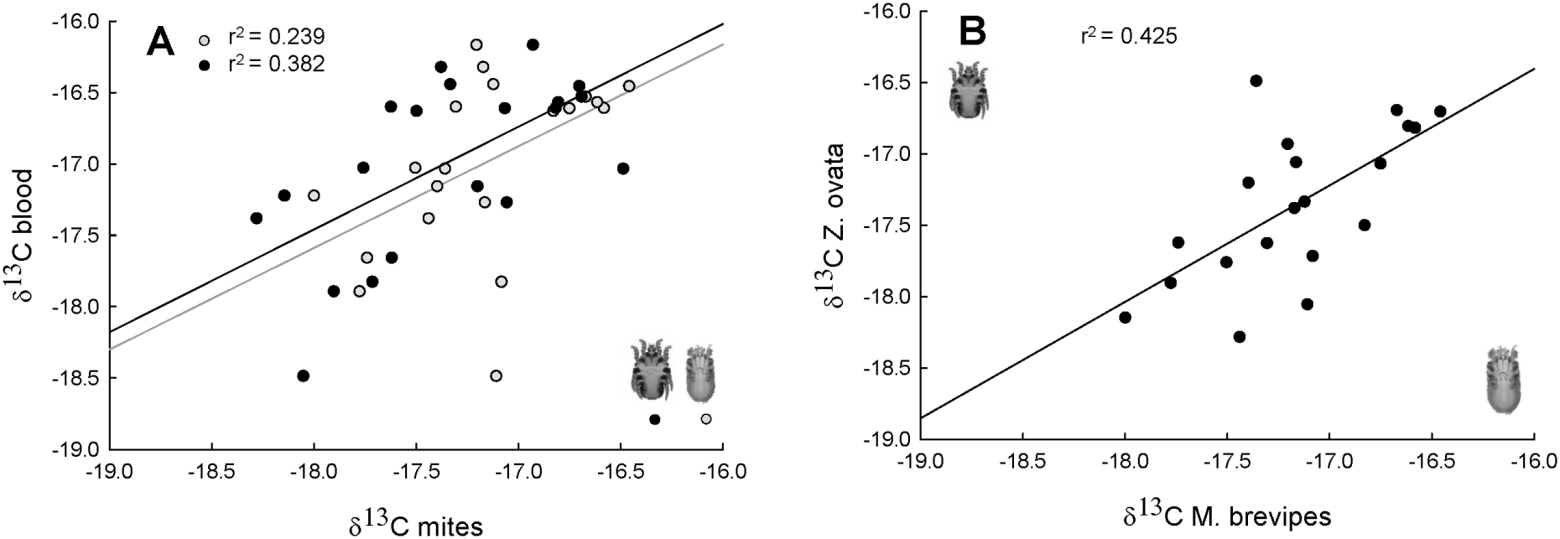
Correlations of carbon isotopic values between each mite species (*Mb* in grey circles and *Zo* in black circles) and host blood (A), and between *Mb* and *Zo* inhabititng the same individual host (B), for 20 Cory´s shearwaters sampled in Veneguera.

We also found a significant correlation in carbon isotopic values between each mite species and host blood (Pearson correlation coefficient, r_(18)_ = 0,489; P = 0.029 for *Mb* and r_(18)_ = 0,618; P = 0.004 for *Zo*, respectively) (Fig 5A) and between the two mite species inhabiting the same host (r_(18)_ = 0,652; P = 0.002) (Fig 5B) and in nitrogen isotopic values between *Zo* and P9-P10 feathers (r_(17)_ = 0,746; P = 0) (S3 Fig), but the latter value may have arisen from a type I error. These results suggest that feather mite diet is mainly based on shared host-associated resources.

## Discussion

In the present study, we investigated the spatial distribution and trophic structure of two dominant and morphologically specialized feather mite species, *M*. *brevipes* and *Z*. *ovata*, inhabiting the flight feathers of Cory´s shearwaters; to determine whether these mites share the same habitats and food resources, i.e. niche partitioning, and whether inter-species competition for these resources is driving these patterns.

### Feather mite spatial distribution

Cory’s shearwaters harbour at least nine feather mite species (L. M. Stefan personal observations), some inhabiting flight feathers, whereas others are restricted to contour feathers. The primary feathers of the Cory´s shearwater breeding in Veneguera, Canary Islands, are mainly inhabited by the two vane-dwelling mite species examined in this study.

The two studied species appear clearly segregated across the wing primary feathers of the host, with *Mb* mainly inhabiting the central primaries (P3-P7) and *Zo* mostly restricted to external primaries (P9-P10). In most of the published studies of within-host distribution of feather mites, the highest mite concentrations have been observed on central primary feathers, with low densities or absence on outer primaries and avoidance of first secondary feathers [12,19,21]. In the present study, the distribution of *Mb* followed this general pattern (concentration on central primaries), but for *Zo* the highest concentrations were found in the outermost two primaries (P9-P10). Our results regarding *Zo* are consistent to some extent with the distribution of *Zachvatkinia caspica* in the Caspian Tern primaries (*Hydroprogne caspia*) [53]. The plumage-space occupied by feather mites could be wider than estimated if the juvenile stages, whose distribution we could not assess by eye, occupy different regions of feathers or wings than do the adults. However, determining the niche occupied by juvenile stages would only be possible by destructive sampling (e.g. removing main primary feathers), which is not possible for a protected species such as *Calonectris borealis*. Contrary to the differences observed in spatial distribution among primary feathers, the two mite species studied here display similar habitat preferences at the within-feather scale. Both species seem to prefer the proximal and distal posterior vane (DPV and PPV regions) and to avoid the proximal anterior vane (PAV).

Several factors may be responsible for microhabitat selection in feather mites. As vane-dwelling feather mites normally inhabit the ventral surfaces of the barbs, barb size and spacing may be of primary importance [19,53]. Both species analyzed in this study have a flattened and heavily sclerotized body, but *Mb* individuals are smaller (adult length around 330 μm and width around 230 μm; measurements from present study) than *Zo* (adult male length around 670 μm and width around 420μm; adult female length around 420 μm and width around310μm; measurements from present study). These differences in body size could be due to species-specific habitat preferences. During fieldwork we observed that both species of mites usually lie along the rachis of the feather, and do not occupy the sides of the barbs (except in highly parasitized hosts), suggesting that the body length should match the interbarb width. But, the primary feathers most commonly occupied by the two mite species exhibited very similar interbarb widths (S4 Fig and S1 Text) and, therefore, interbarb space cannot explain observed differences in spatial segregation.

Another factor that may influence mite distributions could be air turbulence during flight. Due to the strong aerodynamic forces acting over the most external wing feathers, mites seem to avoid this wing region and prefer the median wing region, which may provide additional protection against wind turbulence and feather friction [19]. This is consistent with the distribution of the smaller *Mb* mainly on central primaries, but it does not explain however occupancy patterns of P9 and P10 by *Zo*, which seems associated with some specific morphological traits, in particular their leg structure. *Zo* possesses more separated and elongated forelegs and more laterally inserted hind legs than *Mb*, possibly allowing them to withstand the strong air movement over the outer primaries. The mite distribution can also be influenced by the grooming behavior (preening and scratching) of birds and a number of studies had reported the role of bill and claw morphology for controlling parasites [54,55]. However, this could only be tested with manipulative experiments that alter the bird's ability to preen, experiments that are difficult to apply on seabirds.

The pattern of segregation observed between *Mb* and *Zo* could also be induced by past and/or current competition. Some indirect evidence has been reported on two mites inhabiting kittiwakes *Rissa* spp [19], and on three feather mite species inhabiting the flight feathers of common sandpiper *Actitis hypoleucos* [56]. In both cases, however, distribution data was partially obtained from unrelated host species. In addition, a recent study on two feather mite species inhabiting migratory and sedentary European blackcaps, *Sylvia atricapilla*, showed that mite distribution was primarily influenced by intrinsic, species-specific habitat preferences rather than interactions between the mite species; however, the authors found some evidence of interspecific competition when both mite species occurred on the same sedentary host individuals [25]. In the present study, the two mite species were generally segregated across the primary feathers and showed species-specific morphology, suggesting microhabitat adaptations from past competitive exclusion. However, there was also partial spatial overlap between the two species on P6-P9 feathers, which indicates potential for current habitat competition. Indeed, the distribution of *Mb* on the four birds harbouring only this mite species was slightly displaced towards the outermost primaries, usually occupied by *Zo*, in comparison with the distribution of *Mb* on birds sharing the two mite species (Fig 2 and S1 Fig). Likewise, under current competition, we would expect the abundance of one species to negatively affect the abundance of the competing species. Our results agree with this prediction when both species were present on the same feather, they showed lower overall numbers than when the same feather was occupied by a single species. Similarly, niche overlap among feather regions decreased when both species were present on the same feather. Moreover, the average coefficient of abundance based on individual counts, showed a negative relationship between the two species, that is, high counts of one species were associated with low counts of the other. In general, *Zo* appears as a stronger competitor than *Mb*, except for the PPV region where there was a stronger reduction of *Zo* abundance in the presence of Mb. Overall, these findings clearly support current competition as a factor shaping the distribution and abundance of feather mites within hosts.

### Isotopic signatures of feather mites

Another way mites can diversify their niche to reduce competition is by consuming different food resources. To date, however, the feeding preferences and diet of feather mites remains largely unstudied. In our study, we used SIA analyses to investigate whether the two target species overlap in diet. Our study is the first to examine the trophic structure of feather mites using this method. Both feather mite species, *Mb* and *Zo*, exhibited similar carbon and nitrogen isotopic values. Likewise, the carbon signatures between the two species inhabiting the same individual host were highly correlated, suggesting similar dietary niches. This finding, together with the fact that the two mite species tend to inhabit different primary feathers, indicates that niche partitioning between the two species occurs through spatial rather than trophic segregation.

Here, we considered four possible food items for the mites: blood, skin or feather remains and preen gland oil. Interestingly, of the different host tissues compared, mite isotopic values matched most closely with host blood. That is, mites showed an enrichment of about 2‰ in nitrogen signatures compared to host blood, a value within the expected range of enrichments in nitrogen observed among consumers and their diets [57], and, of the two host tissues (blood and feathers), only carbon isotopic values from blood matched those of the mites. Moreover, we found isotopic values of mites to be close to the values for fleas, known blood feeders, obtained in a previous study investigating the trophic structure of three louse and one flea species from *Calonectris* shearwaters using SIA [40] (see Fig 4). All together, these results imply that host blood is a major food resource for both mite species. However, several other lines of evidence argue against blood as a direct resource for feather mites. First, the chelicerae of both species have the usual chelate-dentate morphology as those of most feather mites [14] (S5 Fig), which is designed for scraping rather than piercing or sucking. These mites are, therefore, unable to puncture host tissues and are constrained to swallowing liquids or small solid materials attached to the feathers. Second, the examination of several slide-mounted specimens at high magnification showed no evidence of blood in their guts, but rather of clear oily material or small mineral-like fragments (S6 Fig). Finally, these mite species live along the feather rachis, where there is no blood to feed on, and there is no evidence that mites move to the skin of the host at any time.

Previous studies on mites suggested that the exogenous material that adhere to feather barbs (scurf, algae, fungi, bacteria, spores, or pollen grains) is one of the main resources for feather mite species [14,31]. However, this is in marked contrast with our isotopic results, which showed a significant correlation between carbon isotopic values of the mites and those from the blood of its individual host (Fig 5A). This correlation can only be explained if mites feed on some resources directly (e.g. blood, skin or preen gland oil) or indirectly (fleas and lice exuviae or excrements) derived from host tissues. Nevertheless, this does not completely discard the possibility that Cory’s shearwater mites feed to some extent on exogenous material. However, measuring the isotopic ratios of the exogenous material caught in the plumage is virtually impossible to do and this limitation could have influenced our isotopic results.

Apart from exogenous material, skin scales and feather fragments have been found in the mite guts, but they were common only in one feather mite species from herons, *Ardeacarus ardeae* [14,30]. In this study both carbon and nitrogen values of the two species of mites were slightly depleted in relation to host skin and feathers (Fig 4), results which rule out these tissues as major food sources. However, it is important to mention that isotopic values of feather may not be as homogeneous as other tissues, because their isotopic values change according to the food consumed when each feather was grown [57,58]. Indeed, we found different isotopic values for P4-P6 compared to P9-P10, but values of the mite species occurring on each of these groups of feathers did not mirror these differences. So far, our results indicate that both mite species feed on some host tissue generated during breeding period (when both, blood and mites were sampled), but not directly on feathers themselves.

Finally, many authors suggest that preen gland oil (predominantly fatty acids and waxes) smeared onto the feathers to maintain feather condition and impermeability is an important food for feather mites [14,30,31]. Carbon values of mites were too enriched (4.49–4.68‰) in relation to preen gland oil, comparing with those previously reported for feather lice or fleas in relation to the tissues consumed on the same seabird host species [40]. However, the correlation in carbon isotopic values between mites and host blood may suggest carbon is taken from the preen gland oil, since its lipids contain mainly carbon and are deposited in uropygial gland through the blood, while nitrogen may be acquired from some exogenous material (i.e. bacteria, algae or fungi).

## Conclusions

In this study, we examined the spatial and trophic segregation of feather mites co-occurring in a seabird host, as well as the role of interspecific competition in explaining these patterns. Our results on spatial niche partitioning showed that the two mite species occupy clearly distinct regions in flight feathers: *Zo* occurs mainly in the outermost primaries and *Mb* in the intermediate primaries and this pattern results from a combination of microhabitat adaptations and ongoing competition. Regarding trophic segregation, our results on mite diet indicated that the two feather mite species show little trophic niche partitioning and likely share the same host food resources, probably preen gland oil, complemented with some exogenous food resources. These results support the prediction that spatial partitioning can only occur when feather mites share the same food requirements. We also show that although past microhabitat specialization may have led to specific morphological differences between the two feather mites allowing them to inhabit different feathers, current interference competition is still playing an important role in shaping the spatial community structure of feather mites. This study also opens new and exciting research perspectives on the trophic ecology of feather mites, calling into question the impact of these arthropods on their host. Our diet results are however preliminary and should be further confirmed and refined using next generation sequencing approaches and fatty acid analyses to identify specific food items in the mite gut. Finally, by combining spatial and trophic approaches in co-occurring seabird feather mites, our work illustrates how symbiotic infracommunities offer excellent models to obtain replicate communities and test niche partitioning hypotheses.

## Supporting Information

S1 Fig Distribution of *Microspalax brevipes* in the primary feathers of Cory´s shearwater left wing for four birds harbouring only this mite species (light gray) and for the 56 birds harbouring both mite species (dark gray). Feathers are ordered following their position in the wing from internal (P1) to external (P10) primary feathers.
(TIFF)

S2 Fig Distribution of *Microspalax brevipes* in the six feathers of Cory´s shearwater left tail. Feathers are ordered following their position in the tail from internal (R1) to external (R6) feathers. “Number of mites” represents the mean number of mites of each species per feather. The 95% confidence limits were computed by resampling using 500 bootstraped values.
(TIFF)

S3 Fig Correlations of nitrogen isotopic values between *Z*. *ovata* and P9-P10 feathers for 19 Cory´s shearwaters sampled in Veneguera. For one bird we did not sampled P9-P10 feathers.
(TIFF)

S4 Fig Interbarb width across all ten primaries for each of the four feather regions. The boxplots correspond to the primary feathers, which are ordered following their position in the wing from internal (P1) to external (P10) feathers (from left to right). The interbarb width was measured on four dead birds. Error bars represent standard error. DPV = distal posterior vane; PPV = proximal posterior vane; DAV = distal anterior vane; PAV = proximal anterior vane. Note that mites were not counted in the DAV and PAV regions of the P10 due to structural features of this feather.
(TIFF)

S5 Fig *Microspalax brevipes* (A) and *Zachvatkinia ovata* (B) chelicera. (TIFF)

S6 Fig Gut content of a *Zachvatkinia ovata* female from Cory´s shearwater showing a detritus bolus of small mineral fragments. (TIFF)

S1 Table *Microspalax brevipes* and *Zachvatkinia ovata* counts on the ten primaries (P1-P10) and six rectrices (R1-R6) for 60 birds. (XLS)

S2 Table Stable isotopic values obtained for the two feather mite species (*Microspalax brevipes* and *Zachvatkinia ovata)* and host tissues (feather, blood, preen gland oil and wing skin) analyzed in this study. (XLS)

S1 Text Interbarb width measurement. (DOC)
