# Supplementary figures and images for: Niche Partitioning of Feather Mites within a Seabird Host, Calonectris borealis
Source: PLoS One. 2015 Dec 9;10(12):e0144728. doi: 10.1371/journal.pone.0144728 (PMC4682861; doi:10.1371/journal.pone.0144728)

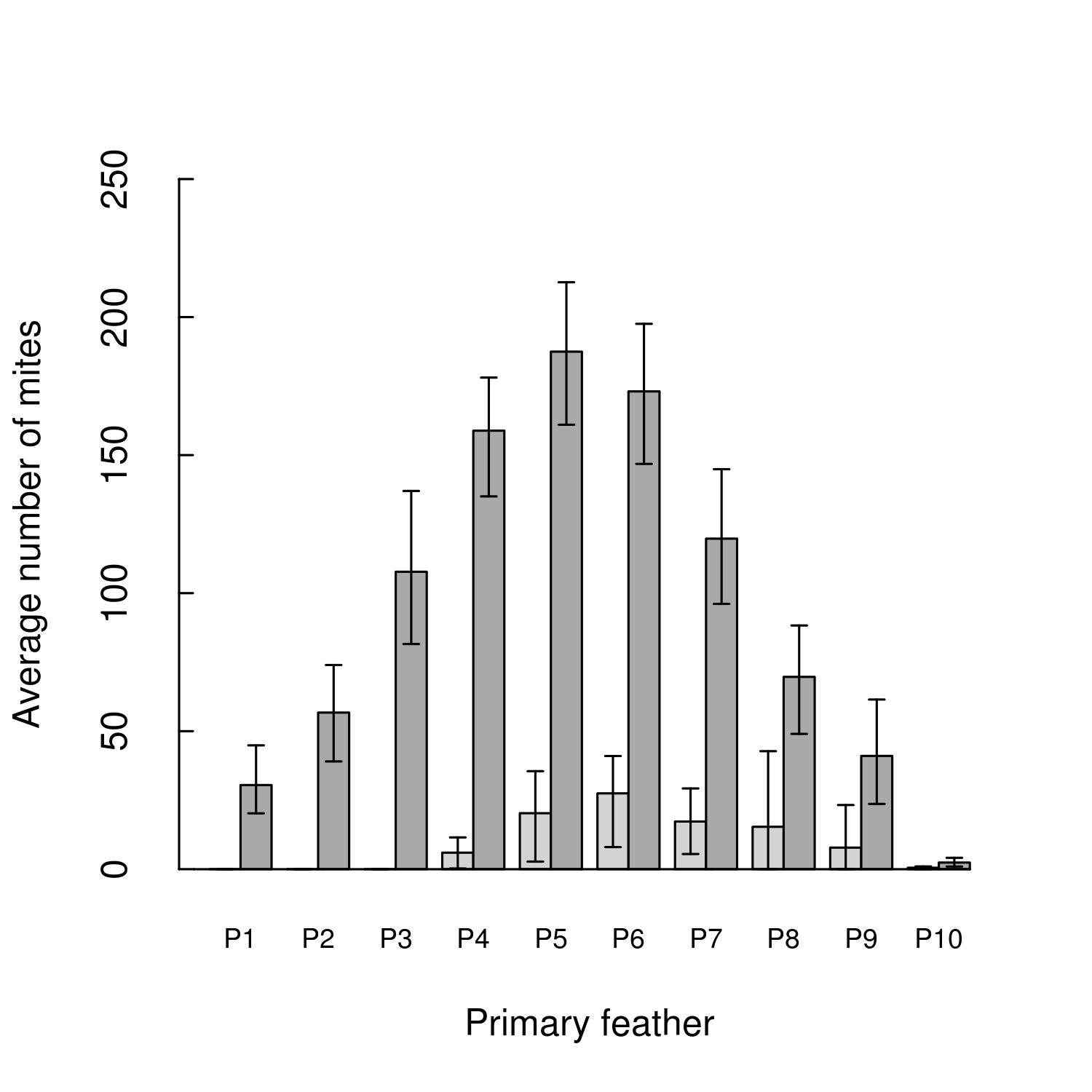

Supplement: S1 Fig — Feathers are ordered following their position in the wing from internal (P1) to external (P10) primary feathers. (TIFF) [file pone.0144728.s001.tiff]

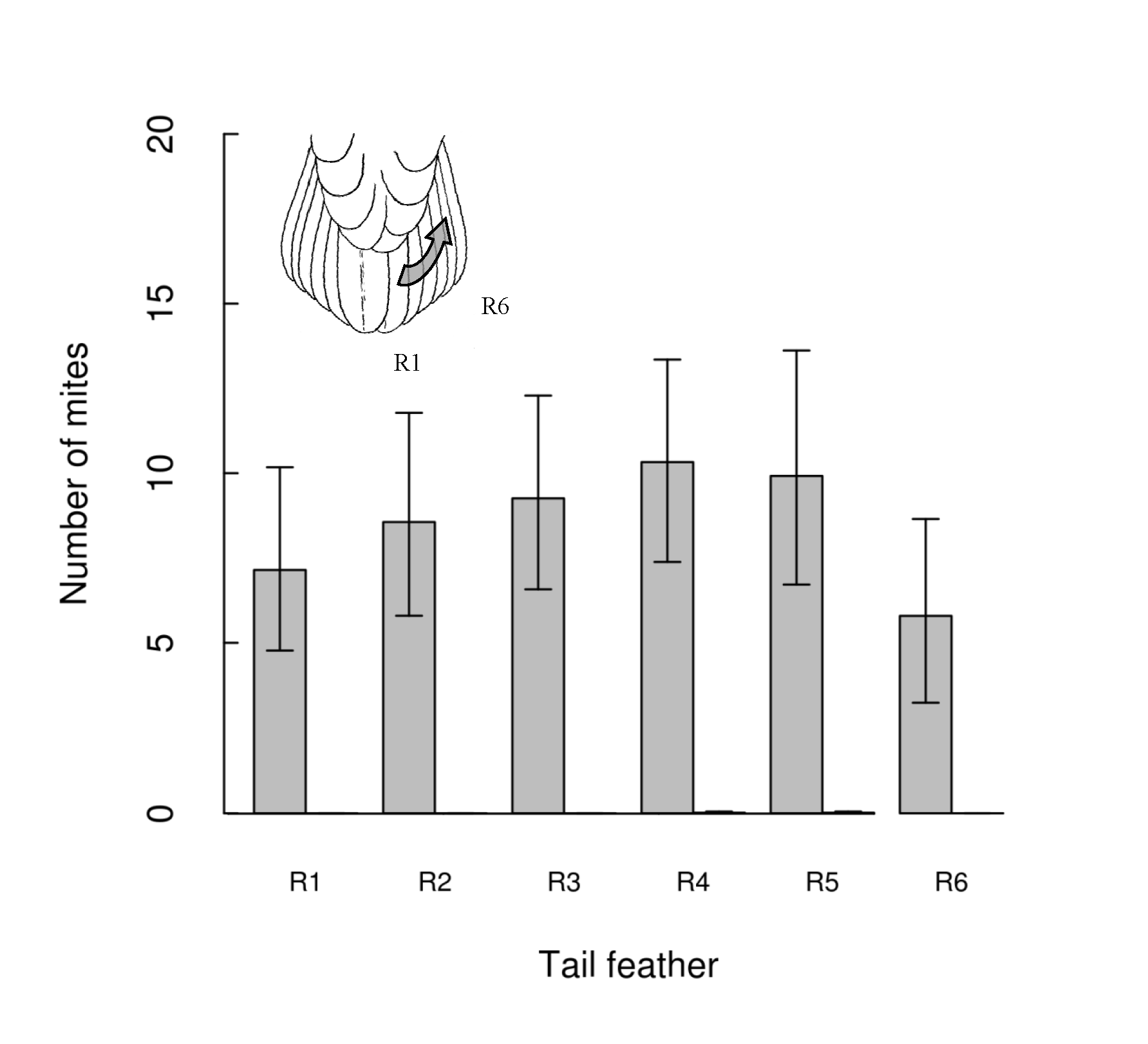

Supplement: S2 Fig — Feathers are ordered following their position in the tail from internal (R1) to external (R6) feathers. “Number of mites” represents the mean number of mites of each species per feather. The 95% confidence limits were computed by resampling using 500 bootstraped values. (TIFF) [file pone.0144728.s002.tiff]

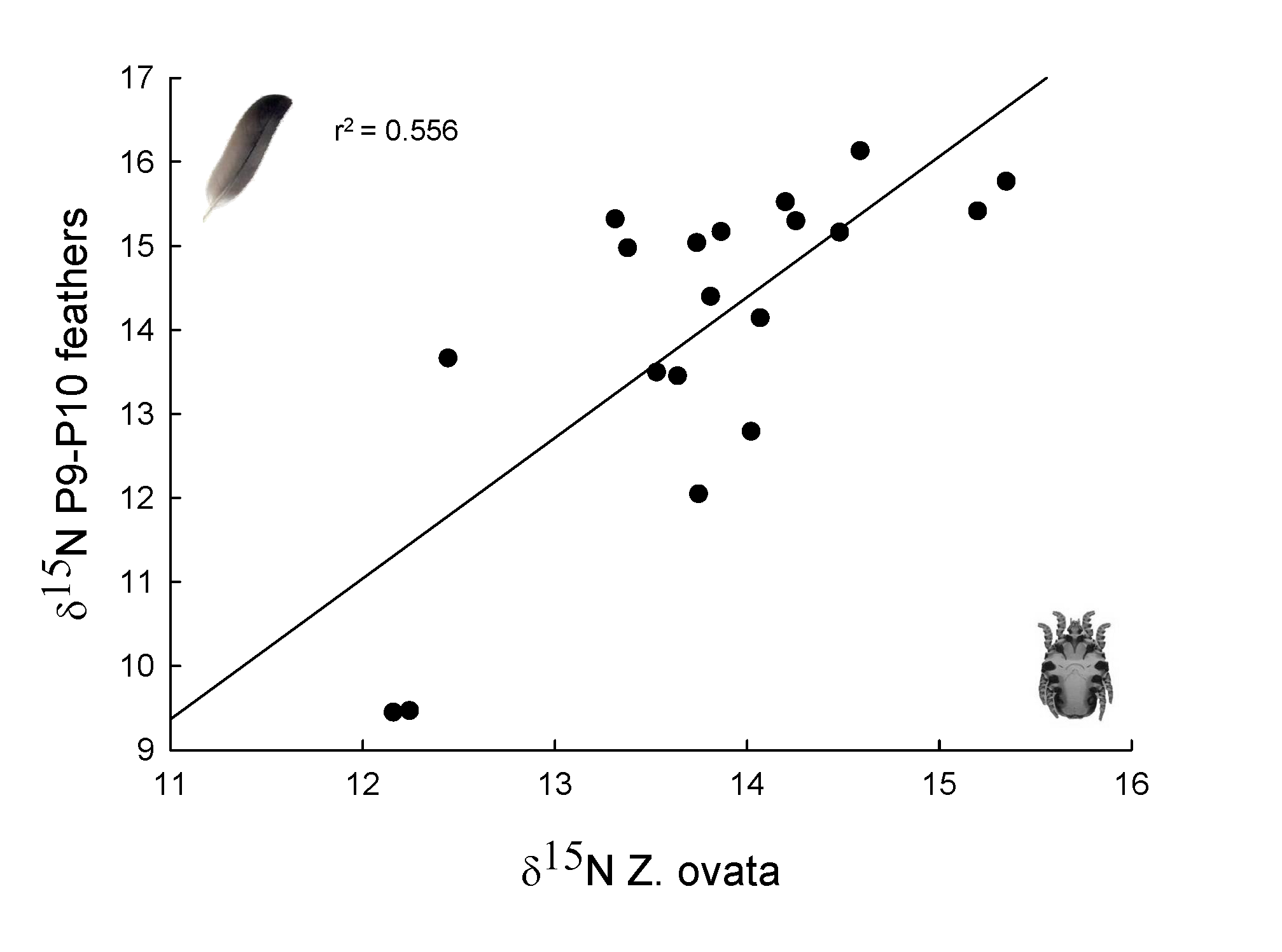

Supplement: S3 Fig — For one bird we did not sampled P9-P10 feathers. (TIFF) [file pone.0144728.s003.tiff]

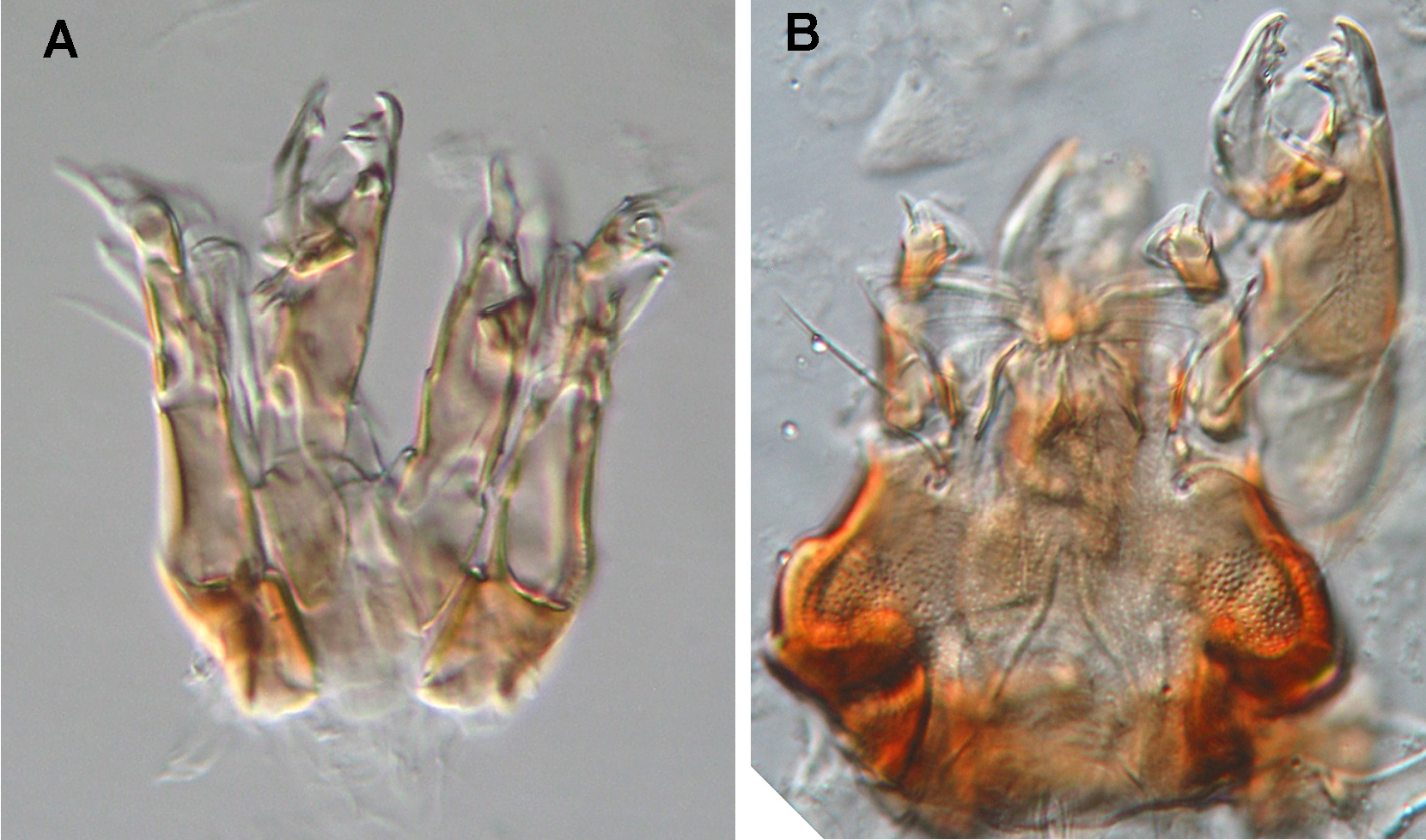

Supplement: S4 Fig — The boxplots correspond to the primary feathers, which are ordered following their position in the wing from internal (P1) to external (P10) feathers (from left to right). The interbarb width was measured on four dead birds. Error bars represent standard error. DPV = distal posterior vane; PPV = proximal posterior vane; DAV = distal anterior vane; PAV = proximal anterior vane. Note that mites were not counted in the DAV and PAV regions of the P10 due to structural features of this feather. (TIFF) [file pone.0144728.s004.tiff]

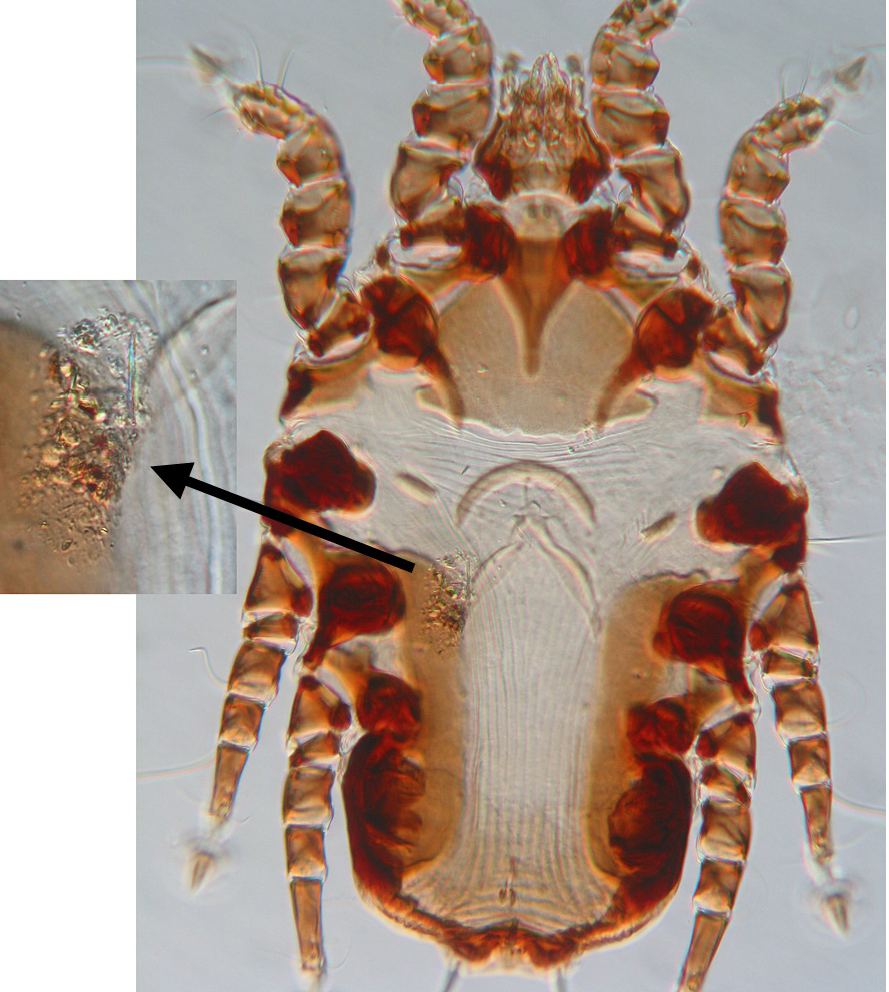

Supplement: S5 Fig — (TIFF) [file pone.0144728.s005.tiff]

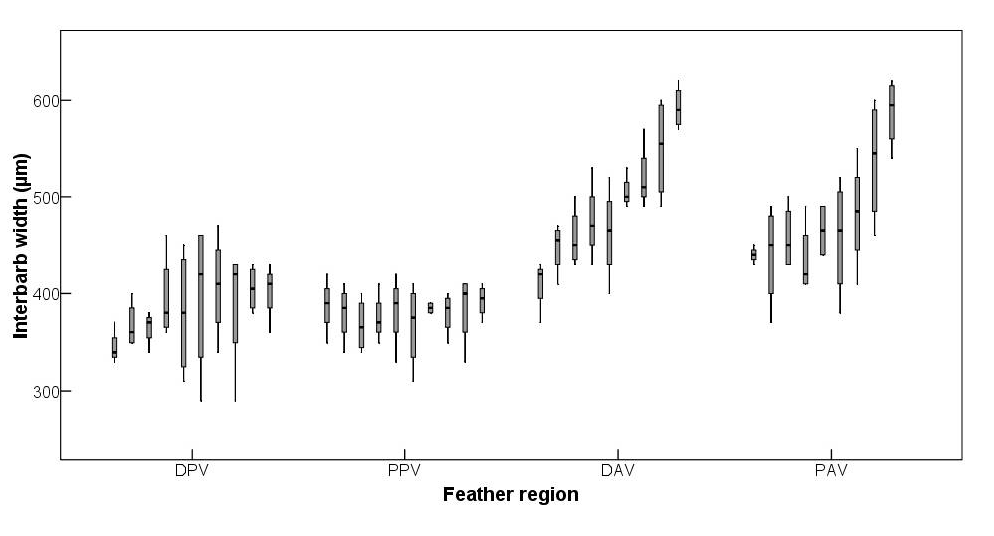

Supplement: S6 Fig — (TIFF) [file pone.0144728.s006.tiff]
