# Supplementary material for: Niche Partitioning of Feather Mites within a Seabird Host, Calonectris borealis
Source: PLoS One. 2015 Dec 9;10(12):e0144728. doi: 10.1371/journal.pone.0144728 (PMC4682861; doi:10.1371/journal.pone.0144728)
Supplement: S1 Text — (DOC) [file pone.0144728.s009.doc]

**Interbarb width measurement**

During fieldwork we observed that both species of mites usually lie along the rachis of the feather, predicting that the length of their bodies should match the width of the interbarb space. Therefore, we measured the interbarb width from photographs of primaries (from dead birds belonging to the same species and from the same island location) to determine how much this varied among different primaries and along the length of a given primary feather, and to check whether this could be related to mite distribution. The interbarb width was measured in each of the four feather regions for all ten primaries in four birds using an Olympus SC30 camera for light microscopy adapted to an Olympus SZX10 stereo microscope. In each feather region, eight consecutive interbarb widths were measured and averaged.

The interbarb width measurements showed no significant differences among feather regions and among primary feathers (Supporting Information Fig. S4). DPV and PPV regions, which are preferred by both feather mite species, exhibited very similar interbarb widths. In PAV and DAV regions, interbarb widths appeared to be slightly higher on the outermost primaries, but also showed the lowest number of mites.
